# Shengui Sansheng San alleviates the worsening of blood–brain barrier integrity resulted from delayed tPA administration through VIP/VIPR1 pathway

**DOI:** 10.1186/s13020-025-01079-0

**Published:** 2025-03-18

**Authors:** Jiacheng Hu, Yiyang Li, Xingping Quan, Yan Han, Jinfen Chen, Mengchen Yuan, Ying Chen, Manfei Zhou, Enze Yu, Jiahao Zhou, Dawei Wang, Ruibing Wang, Yonghua Zhao

**Affiliations:** 1 State Key Laboratory of Quality Research in Chinese Medicine, Institute of Chinese Medical Sciences, University of Macau, Taipa, Macao SAR, People’s Republic of China; 2 Department of Mechanical and Automation Engineering, The Chinese University of Hong Kong, Hong Kong SAR, People’s Republic of China; 3 College of Pharmacy, Shenzhen Technology University, Shenzhen, People’s Republic of China; 4 School of Health Economics and Management, Nanjing University of Chinese Medicine, Nanjing, Jiangsu People’s Republic of China; 5 The First Affiliated Hospital of Guangzhou University of Chinese Medicine, Guangzhou, Guangdong People’s Republic of China; 6 Department of Pharmaceutical Sciences, Faculty of Health Sciences, University of Macau, Taipa, Macau SAR, People’s Republic of China

**Keywords:** Acute ischemic stroke, Blood brain barrier, Shengui Sansheng San, Tissue plasminogen activator, VIP/VIPR1

## Abstract

**Background:**

Intravenous tissue plasminogen activator (tPA) is currently the only FDA-approved thrombolytic therapy for acute ischemic stroke (AIS), however, relative narrow therapeutic time window (within 4.5 h of AIS onset) and high risk of hemorrhagic transformation due to blood–brain barrier (BBB) disruption limit tPA therapeutic benefits for patients. In this study, we extended the time window of tPA administration (5 h after the occurrence of AIS) and investigated whether Chinese medicine classical formula Shengui Sansheng San (SSS) administration was able to alleviate BBB integrity worsening, and the mechanism was related to vasoactive intestinal peptide (VIP)/ VIP receptor 1 (VIPR1) pathway.

**Methods:**

SSS was extracted using aqueous heating method and SFE-CO_2_ technology, and quality control was performed using UHPLC/MS analysis. Male C57BL/6 mice were suffered from middle cerebral artery occlusion (MCAo), followed by the removal of a silicone filament after 5 h, then, t-PA was administered via tail vein injection at once, along with SSS administration by gavage. Hemoglobin levels and Evans blue leakage were measured to assess brain hemorrhagic transformation and BBB permeability, respectively. Transmission electron microscope (TEM) was utilized to present brain microvascular endothelial cells (BMECs) tight junction morphology. TTC staining and laser speckle contrast imaging were employed for infarct volume and cerebral blood flow measurements. The modified neurological severity score (mNSS) test was conducted to evaluate neurological function. The expressions of VIP, VIPR1, ZO-1, Occludin, Lectin, GFAP, NeuN were detected by immunofluorescence staining or western blotting. In vitro, bEnd.3 and N2a cells were insulted by oxygen–glucose deprivation (OGD), and VIPR1 siRNA, and VIP shRNA transfection were respectively performed, and the molecular docking was applied to verify the SSS in-serum active compounds interacted with VIPR1. The transwell system was utilized to detect OGD-insulted BMECs permeability.

**Results:**

SSS treatment significantly reduced the infarct area, cerebral hemorrhage, and neurological deficits, and enhanced cerebral blood flow in AIS mice received intravenous tPA beyond 4.5 h time window. Simultaneously, the permeability of BBB declined, with increased expressions of tight junction proteins ZO-1, and Occludin and proper BMECs tight junction morphology, and it suggested that VIP was released by neurons rather than astrocytes or BMECs. It also showed high expressions of VIP and VIPR1 in the penumbra area. The inhibition of VIP in N2a cells or VIPR1 in bEnd.3 cells abolished the viability and integrity of OGD-insulted bEnd.3 cells treated by tPA after SSS-containing serum administration, and the SSS in-serum active compounds were proved have high affinity to VIPR1 by molecular docking.

**Conclusion:**

SSS alleviates the worsening of BBB integrity resulted from delayed tPA administration, reduces hemorrhagic transformation and infarction volume, and ameliorates brain blood flow and neurological function in AIS mice. The mechanisms are associated with the activation of VIP/VIPR1 pathway to enhance BMECs viability and maintain tight junction phenotype.

**Graphical Abstract:**

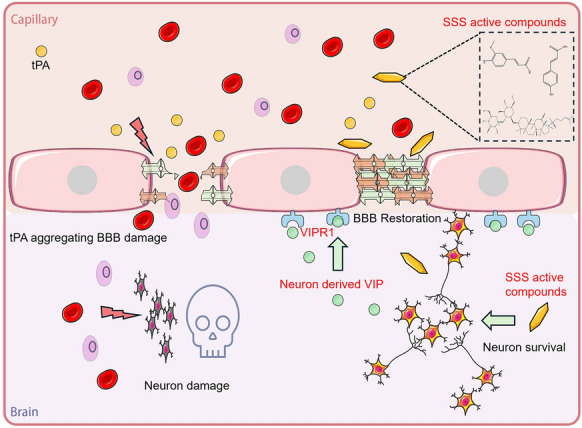

**Supplementary Information:**

The online version contains supplementary material available at 10.1186/s13020-025-01079-0.

## Introduction

As the most common type of stroke, ischemic stroke (IS) accounts for approximately 87% of all strokes, and is one of the leading causes of death and long-term disability worldwide due to thrombus impeding focal blood flow perfusion resulting in blood–brain barrier (BBB) disruption and irreversible neurons injury [1, 2]. Around 9.1 million people suffer from IS every year [3]. Tissue plasminogen activator (tPA) is a thrombolytic medication employed for acute IS (AIS) patients by converting plasminogen into plasmin for recanalization [4, 5]. Since the U.S. FDA approved tPA as the thrombolytic drug for AIS treatment in 1995, it has become one common and standard therapy recommended by diverse guidelines [4]. The time of intravenous tPA administration is restricted within 4.5 h of the onset of symptoms, which maximizes the efficacies and minimizes the risks [5, 6]. Otherwise, beyond 4.5 h administration, the therapeutic effectiveness significantly declines, and the risks of thrombolysis-related bleeding complications arise, particularly within the first 24 h after tPA treatment [7, 8].

BBB disruption is one of the major side effects resulted from delayed tPA administration [9, 10]. Serving as a regulated interface between the peripheral circulation and brain parenchyma, the basic structure of BBB consists of tightly connected brain microvascular endothelial cells (BMECs) [11, 12]. BMECs have specialized tight junction that selectively restricts the permeability of large molecules and water-soluble substances. The tight junction between BMECs is composed of a series of proteins including claudin, occludin, and zonula occludens (ZO), which work together to maintain BBB integrity [13–15].

As a neuromodulator, Vasoactive Intestinal Peptide (VIP), first discovered in intestinal, is a bioactive peptide widely presented in mammals, and with 72-amino-acid peptide exerts multiple functions including vasodilation, gastrointestinal regulation, and influencing the activities of immune and nervous systems [16, 17]. Importantly, research conducted in recent years revealed VIP plays an important role in maintaining gut barrier [18, 19]. Our previous research also demonstrated that BBB integrity was maintained by enhancing the expression of VIP and its receptors [20].

Shengui Sansheng San (SSS), composed of *Ginseng Radix et Rhizoma*, Cinnamomi Cortex (dried bark of *Cinnamomum verum*), and *Angelicae Sinensis Radix*, originates from the ancient Chinese medicine book entitled "Zhang Shi Yi Tong" and has been used in stroke treatment for over 300 years in China [21]. Our previous research results indicated that SSS treatment not only reduced the infarct area in rats with middle cerebral artery occlusion (MCAo), but also improved focal cerebral blood flow and neurological function. These preliminary results provide strong evidence for SSS's role in maintaining BBB integrity after cerebral ischemia. However, it remains unknown whether SSS can attenuate the worsening of BBB integrity resulted from the over-time-windowed tPA therapeutic, and the underlying mechanisms regarding VIP/VIPR1 still need to be validated.

In this study, we investigated the therapeutic effects of SSS on the worsening of BBB integrity after delayed intravenous tPA administration, and discovered the mechanism related to VIP/VIPR1 pathway.

## Materials and methods

### SSS aqueous extract

*Ginseng Radix et Rhizoma*, *Angelicae Sinensis Radix* and *Cinnamomi Cortex* purchased from Guangzhou Zisun Pharmaceutical Co., Ltd., China were identified by Prof. Hua Yu in Institute of Chinese Medical Sciences (ICMS), University of Macau (UM). They were mixed in a 1:1:1 ratio, and then, combined with water at a 1:10 ratio (herb weight to water volume), the mixture was heated by the ZNHW series heating mantle at 260 °C until it reached a gentle boil. Then the heating temperature was reduced to 140 °C, maintaining a continuous boiling state for 40 min. After cooling to room temperature, the mixture was filtered through cheesecloth to remove coarse particulates, and it was further filtered using vacuum filtration. The remaining liquid was concentrated using rotary evaporation to obtain the SSS aqueous extract which was stored at ICMS sample center, UM.

### SSS volatile oil extract

The extract process was conducted using SFE-CO_2_ technology (SFT-250, Supercritical Fluid Technologies). Briefly, 300 g of sample was measured and placed in an extractor. Extractions were performed at a fixed set of temperature (44 °C), solvent flow rate (0.3 kg/h), and extraction time (2 h), while pressure was controlled at 300 bar. The separator conditions were set at 15 bar and 25 °C. Extracts were collected in 50 mL centrifuge tubes, sealed, and kept at 4 °C prior to analysis, and were also stored at ICMS sample center, UM.

### Quality control for SSS

Before quality control, SSS aqueous extract and volatile oil extract were mixed at the ratio of 1000:7 according to the extraction ratio of SSS volatile oil (Table S2), and the SSS mixture was determined as therapeutic medication in the study of animal experiment. Analyses were performed using an Ultra-High Performance Liquid Chromatography (UHPLC, Vanquish, Thermo Fisher Scientific) coupled to Orbitrap Exploris 120 mass spectrometer (Orbitrap, Thermo Fisher Scientific). UHPLC chromatographic separation was conducted using an Acquity UHPLC system (Acquity LC, Waters) installed with a Waters UPLC column (ACQUITY UPLC BEH Amide 1.8 μm, 2.1 × 100 mm, Waters, Milford, MA). The mobile phase A consisted of 25 mM ammonium acetate and 25 mM ammonium hydroxide in water, and the mobile phase B consisted of 100% ACN. The flow rate was set at 0.5 mL/min. The injection volume was 2 μL, and the samples were maintained at 4 °C in the autosampler. The Orbitrap Exploris 120 mass spectrometer was used for its ability to acquire MS/MS spectra on information-dependent acquisition (IDA) mode in the control of the acquisition software (Xcalibur, Thermo). In this mode, the acquisition software continuously evaluated the full scan MS spectrum. The ESI source conditions were set as following: sheath gas flow rate as 50 Arb, Aux gas flow rate as 15 Arb, capillary temperature 320 °C, full MS resolution as 60,000, MS/MS resolution as 15,000, collision energy: SNCE 20/30/40, spray voltage as 3.8 kV (positive) or −3.4 kV (negative), respectively.

### MCAo model establishment, group division and administration

The animal experiment protocol was approved by the Ethics Committee of the University of Macau (APP-ARE-012). The animal study was conducted according to the Guide for the Care and Use of Laboratory Animals (8th edition, Washington, D.C.: The National Academies Press, 2011). Male C57BL/6 wild type mice (8–10 weeks, 20–25 g) were anesthetized with an intraperitoneal injection of 1.25% (w/v) tribromoethanol before surgery. The MCAo model was established according to our previous work [24]. Briefly, an incision was made in the neck of the mice to isolate the common carotid artery, internal carotid artery, and external carotid artery. The external carotid artery was temporarily ligated, and a silicone-coated monofilament was inserted approximately 1.6–1.8 cm from the micro-incision in common carotid artery, advancing towards middle cerebral artery via internal carotid artery. After modeling, the incision was sutured and disinfected. The mice were kept warm at 37 °C using a heating pad to prevent loss of body temperature. Food and water were provided ad libitum post-surgery. To allow reperfusion, the suture was withdrawn 5 h after MCAo and the incision was re-sutured. All mice were randomly divided into five groups: SHAM, MCAo, tPA, SSS, and tPA + SSS (T + S). Based on the results of preliminary experiment (Fig. S1A), the SSS mixture including aqueous extract (100 mg/kg) and volatile oil (0.7 mg/kg) was administered once before surgery and treated again at 5 h post-surgery by gavage. tPA was dissolved in sterile water for injection and administered via the tail vein at a dose of 10 mg/kg, 5 h after surgery.

### TTC staining

Twenty-four hours after treatment, the mice were deeply euthanized and sacrificed before perfused with PBS. The brains were quickly removed and sliced into four continuous 2 mm thick coronal sections, and were incubated in 2,3,5-Triphenyltetrazolium chloride (TTC) for 10 min in the dark at 37 °C. Photographs of the slices were taken, and ImageJ software (Version 1.6, ImageJ bundled with 64-bit Java 8) was used to quantify the infarct volume.

### Cerebral blood flow evaluation

We conducted the cerebral blood flow evaluation according to Zhang’s research [22]. Briefly, mice were anesthetized with tribromoethanol 1.25% (w/v), and their skulls were exposed to delineate regions of interest on both the ipsilateral and contralateral sides of the infarct sites. Laser speckle contrast imaging (LSCI) was performed using a 785 nm laser with a maximum output power of 70 mW. Imaging was carried out 24 h post-stroke.

### BBB leakage evaluation by Evans Blue dying

Evans Blue (EB) extravasation into the ischemic brain was employed to evaluate the severity of BBB leakage. Under anesthesia, mice were injected via the tail vein with 2% EB (3 ml/kg, Sigma); after circulating for 1 h, the mice were perfused with 50 ml of PBS to clear the circulating EB, completely. Brain tissue was then harvested and sliced into continuous 2 mm thick coronal sections. The brain slices were imaged using an in vivo imaging system to determine the permeability extent of BBB. The fluorescence intensity (excitation at 620 nm, emission at 710 nm) of the ipsilateral hemisphere and contralateral hemisphere was measured by IVIS® Spectrum small animal image system (PerkinElmer) to assess the permeability of BBB 24 h after the MCAo establishment.

### Transmission electron microscopy (TEM) imaging

Cerebral microvascular morphology and tight junctions were observed by TEM. Briefly, mice cortex brain tissues were collected and fixed in 2.5% glutaraldehyde solution, then, brain tissue was dehydrated in 30–95% ethanol before penetration and embedding. Ultra-thin sections (60 nm) were prepared using a Leica UC7 ultramicrotome and mounted on 150-mesh formvar-coated copper grids. These sections were stained sequentially with 2% uranyl acetate in ethanol for 8 min, rinsed in 70% ethanol and ultrapure water, then stained with 2.6% lead citrate for another 8 min. Following additional rinsing in ultrapure water and overnight drying, the samples were imaged using TEM (HT7800, Hitachi).

### Cerebral hemorrhage transformation

The hemoglobin detection kit was purchased from Beijing Solarbio Science & Technology Co., Ltd. Brain tissue was homogenized using a grinding pestle and dissolved in PBS. After centrifugation at 1000 rpm for 10 min at 4 °C, the supernatant was collected and measured by a plate reader (Molecular Devices, LLC, FlexStation 3 Multi-Mode Microplate Reader). The absorbance at 400 nm was then measured. The hemoglobin content in each group was determined by comparing the absorbance with the standard curve of the hemoglobin standard samples.

### Assessment of neurological deficits

Neurological deficits in mice were assessed 24 h after MCAo operation. The modified Neurological Severity Score (mNSS) was used for neurological grading. The analysis was conducted by a blinded investigator who was unknown the groups division. The detailed scoring standard was provided in supplement materials (Table S1).

### Immunofluorescence staining

Mice were euthanized and perfused with 50 mL of cold PBS, followed by perfusion with 50 mL of 4% paraformaldehyde (PFA). After perfusion, the brains were carefully removed and fixed in 4% PFA for 24 h. Following fixation, the brains were placed in 15% sucrose solution for 24 h for dehydration, then transferred to 30% sucrose solution for an additional 24 h. After dehydration, the brains were placed in a specific mold, embedded in OCT compound, and frozen overnight at −80 °C. Using a cryostat, the brains were sectioned into 20 μm thick slices and mounted onto slides. The brain sections were fixed in 4% PFA, permeabilized with 0.3% Triton X-100, and blocked with blocking solution for 1 h. The primary antibody ZO-1 (1:250, Proteintech, 21773-1-AP), Occludin (1:250, Invitrogen, 71-1500), VIP (1:100, Invitrogen, PA5-78224), VIPR1 (1:250, Abcam, ab139746), GFAP (1:400, CST, 3670T), NeuN (1:400, CST, 94403T) was incubated overnight at 4 °C, followed by incubation with the corresponding secondary antibodies (Alexa Fluor® 488 or 594 of rabbit or mouse, 1:500, Abcam, ab150113; ab150080; ab150077) at room temperature for 1 h. Lectin (1:100, VectorLabs, DL-1174-1) was incubated at room temperature for 1 h. Finally, after nucleus staining by DAPI (C1005, Beyotime), fluorescence signal was observed by using fluorescent microscope (Lecia, DMi8), and mean fluorescent intensity was measured by ImageJ software (Version 1.6, ImageJ bundled with 64-bit Java 8).

### Primary culture of neurons

Primary neurons were isolated from the cerebral cortices of P0 C57BL/6 mice. After euthanizing the mice on ice, the brains were removed, and the cortices were dissected in starve DMEM medium. The tissue was then dissociated by incubation with papain digestion solution (2 mg/mL papain in PBS) at 37 °C for 15 min, followed by gentle trituration. The digestion was terminated with warm Neurobasal medium containing 10% fetal bovine serum (FBS), and the cell suspension was centrifuged at 1000×*g* for 5 min. The pellet was resuspended in Neurobasal medium supplemented with 2 mM l-glutamax-I Supplement, B27, and passed through a 70 µm strainer. The suspension was plated onto poly-d-lysine-coated plates at 1–2 × 10^6^ cells/mL and incubated at 37 °C for 4 h, after which the medium was replaced with fresh Neurobasal with B27. Neurons were cultured for 7–10 days, with medium replaced every 2–3 days.

### Western blotting

Proteins were extracted from the cortex of ischemic ipsilateral hemisphere in mice. The brain tissue was lysed using RIPA lysis buffer. For protein sample preparation, grinding beads were added to the tissue, and homogenized, followed by protein concentration determination using the BCA assay. After quantification, proteins were separated by SDS-PAGE (10 µl per lane) and transferred to a PVDF membrane. The membrane was blocked with 3% BSA at room temperature for 1 h. The bands were incubated overnight at 4 °C with a primary antibody against Actin (1:10,000, Proteintech, 66,009–1-Ig), ZO-1 (1:1000, Proteintech, 21773-1-AP), Occludin (1:500, Invitrogen, 71-1500), VIP (1:500, Invitrogen, PA5-78224), VIPR1 (1:1000, Abcam, ab139746) diluted according to the manufacturer's instructions, followed by 1 h incubation with a secondary antibody against Anti-Mouse HRP (1:3000, Abcam, ab97023), Anti-Rabbit HRP (1:3000, Abcam, ab6721) at room temperature. Protein bands were detected by using a ChemiDoc MP imaging system (BIO-RAD), and quantification was performed by using ImageJ software (Version 1.6, ImageJ bundled with 64-bit Java 8).

### SSS-containing serum development

SSS-containing serum was prepared by orally administering SSS mixture including aqueous extract (100 mg/kg) and volatile oil extract (0.7 mg/kg) twice daily for 7 consecutive days. Three hours after the last dose was administered, the mice were anesthetized, and blood was taken from orbital venous sinus. The peripheral blood was put at room temperature for 2 h to allow clotting, followed by centrifugation at 3000 r/min, 4 °C for 10 min. The supernatant was then carefully collected and filtered through a 0.22 μm membrane. The filtered serum was inactivated at 56 °C for 30 min. The prepared serum was stored at −80 °C until further use.

### Oxygen glucose deprivation

The medium of bEnd.3 and N2a cells were replaced with glucose-deprived DMEM, and they were transferred to a hypoxic chamber (MIC-101, Billups-Rothenberg). The oxygen percentage in the chamber was determined by Nuvair O_2_ QuickStick (Nuvair). The ventilation rate of N_2_ was controlled between 15 and 20 L/min, and the chamber was sealed until the O_2_% ≤ 0.5%, and then cells were cultured at 37 °C for 2.5 h.

### Cell viability assay (CCK8)

1 × 10^5^ bEnd.3 cells seeded into 96 well plate were insulted by OGD, and the medium in each well was aspirated and discarded. CCK8 reagent (C0037, Beyotime) was then diluted in DMEM complete medium at a 1:10 ratio, and 100 μL of CCK8-containing medium was added to each well. The plate was incubated in a 37 °C, 5% CO_2_ incubator for 2 h. After incubation, cell viability was assessed by measuring the absorbance at 490 nm using a microplate reader (Molecular Devices, LLC, FlexStation 3 Multi-Mode Microplate Reader).

### VIPR1 siRNA transfection

siRNAs were purchased from Tsingke Biotechnology Co., Ltd. bEnd.3 cells were seeded into the upper chamber of 24-well Transwell inserts for 24 h prior to transduction. Lipo2000 reagent was dissolved in Opti-MEM medium and incubated at room temperature for 5 min to ensure complete dissolution. Then, siRNA was added to the mixture and incubated at room temperature for an additional 15 min. The prepared transfection mixture was then added to the cell culture medium. After 24 h, the medium was replaced with fresh DMEM complete medium. Cells were collected 72 h post-transfection and knockdown efficiency was validated by western blotting. The inserted VIPR1 oligos are listed below.

siVIPR1-1 Sequence (5′-3′):

GACUGAGUUCUACGAUGCA (dT)

siVIPR1-2 Sequence (5′-3′):

GGACGAUUGUCAGGAUCCA (dT)

siVIPR1-3 Sequence (5′-3′):

CACUGUACUCGAAACUACA (dT)

### VIP shRNA transfection

Lentivirus was purchased from Tsingke Biotechnology Co., Ltd. The lentiviral vector used was pLVX-shRNA2-Puro-GFP. 5 × 10^5^ N2a cells were seeded in 60 mm culture dishes for 24 h prior to infection, and when they reached 60–70% confluence, the virus was added at a multiplicity of infection (MOI) of 2 × 10^7^ TU/mL, along with 2 μg/mL polybrene, and incubated for 24 h. Subsequently, the culture medium was replaced with fresh DMEM complete medium. After 48 h post-infection, 2 μg/mL puromycin was added for selection. The infection efficiency was assessed by immunofluorescence staining after one round of selection. The inserted VIP oligos are listed below.

shVIP1 Sequence (5′-3′): GATCGGATACTCTTCAGTGTGCTGTTCTCGAGAACAGCACACTGAAGAGTATCTTTTTT

shVIP2 Sequence (5′-3′): GATCGTGGATGACAGGATGCCGTTTGCTCGAGCAAACGGCATCCTGTCATCCATTTTTT

### BMECs permeability assay

The method for assessing BMECs permeability was accordance in our previous research [23]. Briefly, bEnd.3 cells (5 × 10^4^ cells per well) were seeded into the upper chamber of 24-well Transwell inserts with 0.4 μm pores and cultured for 72 h until confluence. Prior to OGD treatment, 2 mg/mL TRITC-Dextran (4.4 kDa, T1037, Sigma-Aldrich) in DMEM and glucose-deprived DMEM were added to the upper chamber in Control and OGD groups, respectively. Glucose-deprived DMEM was added to lower chambers. Then, 20 μg/mL tPA, 10% SSS containing serum, blank serum was added into indicated groups. After OGD 2.5 h, 50 μl medium of each upper and lower chambers was collected, and TRITC-dextran fuorescence intensity was read by microplate reader (SpectraMaxM5) at wavelength of excitation: 550 nm and emission: 572 nm. The permeability coefficient was calculated by the following method as previously reported [24].

$${\text{P}}_{{{\text{dextran}}}} = \left( {{\text{RFU}}_{{\text{lower chamber}}} {\text{/RFU}}_{{\text{upper insert}}} } \right)\left( {\text{1/S}} \right)\left( {\text{V}} \right)\left( {\text{1/t}} \right).$$

“RFU” was the fluorescent intensity of upper insert and lower chamber, and “S” indicated the surface area of cell monolayer, while “V” was the volume of lower chamber and “t” represented the time TRITC-dextran spread.

### Molecular docking

The 3D structure of VIPR1 protein (PDB ID: 1OGT) was downloaded from the RCSB Protein Data Bank and water molecules and polar hydrogen atoms were removed using Pymol (v4.6.0). The structure of 4-Hydroxycinnamic acid, Ginsenoside Rg3, ferulic acid were downloaded from Pubchem. The interaction between these compounds and VIPR1 was analyzed by using the latest version of AutoDock (v1.5.7). We conducted the docking process according to the official user manuals. The grid box used for docking was centered at x center = 8.949, y center = 2.407, z center = 13.38 and number of points in x-dimension = 114, in y-dimension = 86, in z-dimension = 126 with spacing (angstrom) = 0.536. The binding affinity of these compounds to VIPR1 were accessed by the outcoming binding energy. The binding mode was visualized by using Pymol (v4.6.0) and LigPlot + (v2.2.8).

### Statistical analysis

All experimental data were presented as mean ± standard deviation (SD) analyzed by using GraphPad Prism 8 software. Significant differences were determined by one-way analysis of variance (ANOVA) followed by Tukey’s multiple comparisons test, when *p* value < 0.05, the statistical significance was determined.

## Results

### Extraction and quality control results of Shengui Sansheng San

**Fig. 1:**
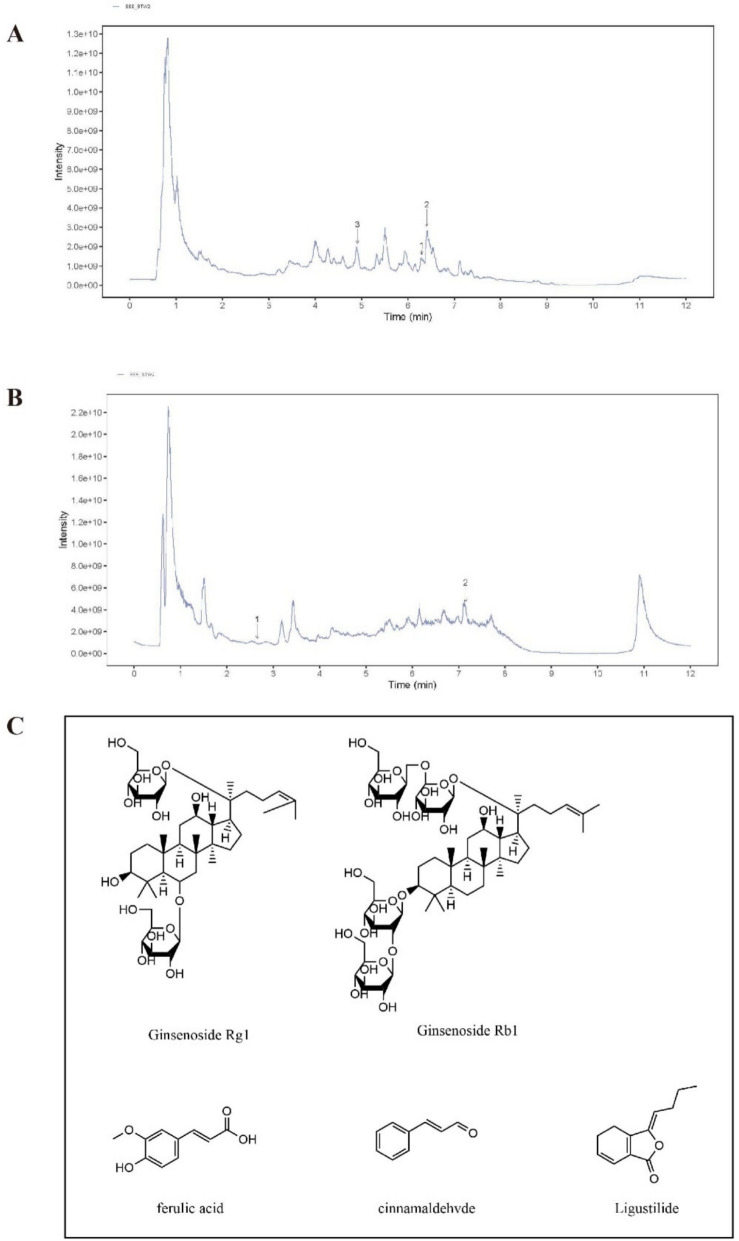
Quality control of Shengui Sansheng San. **A, B** Ultra-High performance liquid chromatography (UHPLC) chromatogram of SSS extraction. A-(1) Ginsenoside Rg1, A-(2) Ginsenoside Rb1, A-(3) ferulic acid, B-(1) cinnamaldehvde, B-(2) Ligustilide. **C** Chemical structure of Ginsenoside Rg1, Ginsenoside Rb1, ferulic acid, cinnamaldehvde, Ligustilide

Before extraction, we grinded ginseng, angelica, and cinnamon into powder and then mix them together in a 1:1:1 ratio. Totally 5926.7 g SSS were used for SSS volatile oil extraction and 42.64 g SSS volatile oil was extracted from SSS powder (Table S2). A representative UHPLC chromatogram of SSS extract was presented in Fig. 1A, B. the corresponding representative components in SSS were Cinnamaldehyde, Ginsenoside Rg1, Ginsenoside Rb1, trans-Ferulic acid, Ligustilide (Fig. 1C).

### SSS treatment alleviated neurological deficits in AIS mice received delayed tPA administration

**Fig. 2:**
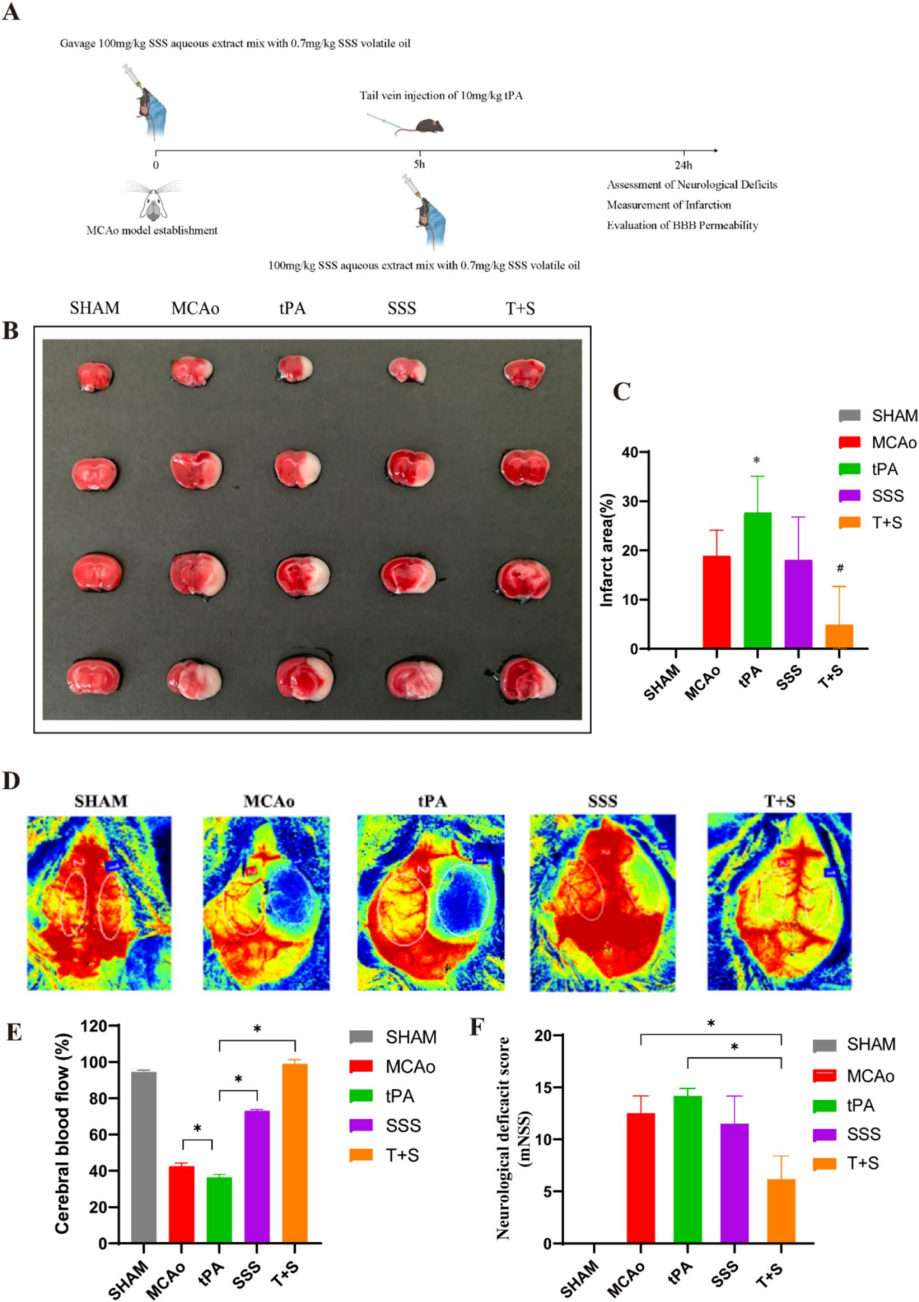
SSS treatment improved therapeutic efficacies in AIS mice after delay tPA administration. **A** Schematic diagram of experimental design. **B** Representative Images of TTC Staining. **C** Quantitative analysis for infarct volume (n = 6). **D** Representative LSCI images of the ipsilateral and contralateral hemispheres. **E** Quantitative analysis of CBF. **F** Modified neurological severity score (mNSS) evaluation. *p < 0.05 vs MCAo, ^#^*p* < 0.05 vs tPA

The experimental administration of flow chart was presented in Fig. 2A. As shown in Fig. 2B, C, the delayed tPA administration failed to improve infarct volume in AIS mice while even expanded it. SSS treatment alone showed ineffective on infarct volume. However, the combined use of tPA and SSS helped reverse the enlargement of the infarct area caused by delayed tPA treatment. Meanwhile, the results indicated that delayed tPA administration led to severe reduction in cerebral blood flow compared with MCAo group. Other than that, the supplement of SSS improved 65% cerebral blood flow in ipsilateral of AIS mice compared to tPA group (Fig. 2D, E). Similarly, the neurobehavioral scoring results showed that delayed tPA administration worsened neurological function, and the combination treatment ameliorated neurological deficits (Fig. 2F).

### SSS treatment maintained the integrity of BBB and reduced hemorrhagic transformation in AIS mice administered by delayed tPA

**Fig. 3:**
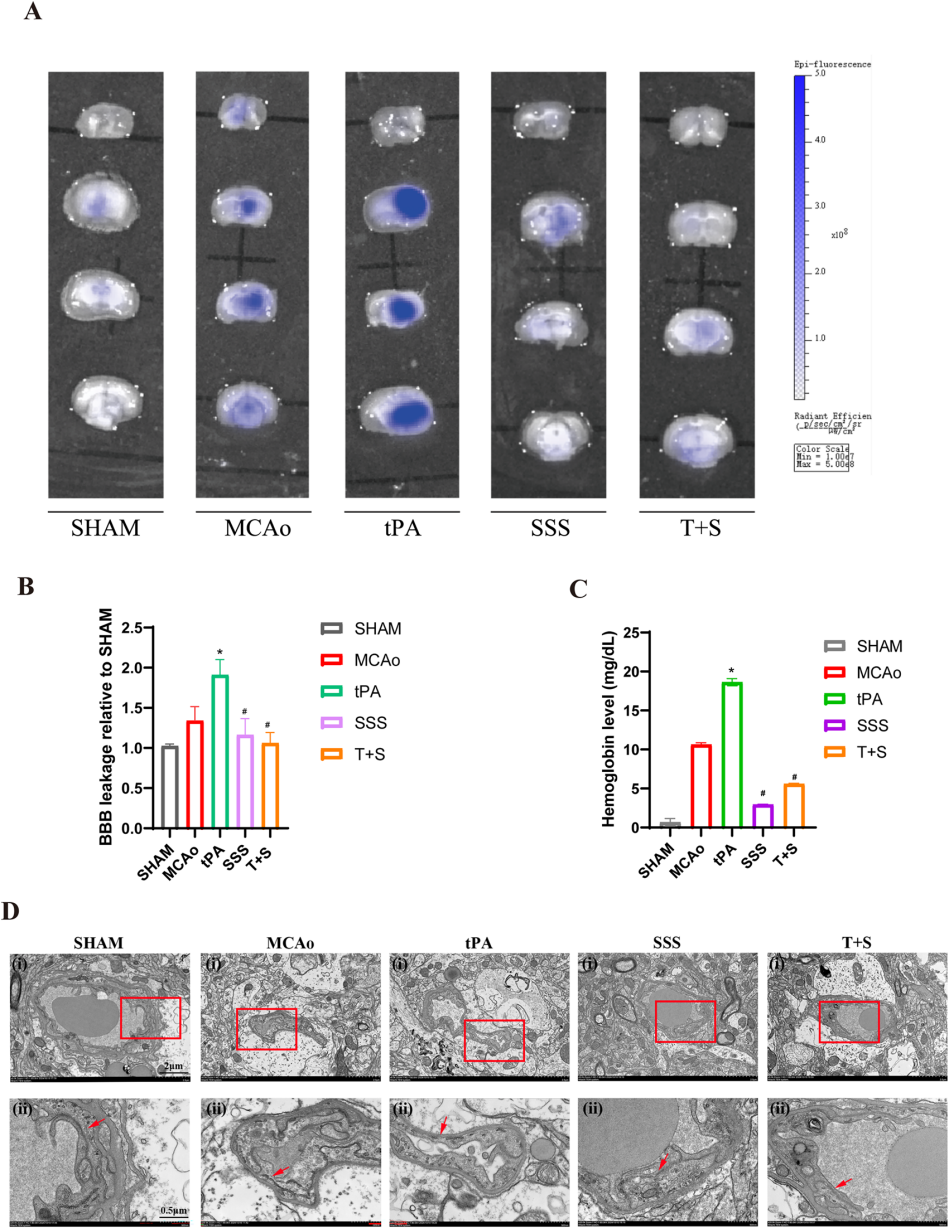
SSS treatment maintained the integrity of blood–brain barrier and reduced the risk of hemorrhagic transformation. **A** Representative image of Evans blue leakage. **B** Quantitative analysis of Evans blue leakage. **C** Quantitative assessment of hemoglobin levels. **D** The TEM images for BBB phenotype (The tight junctions indicated by the red arrows). Upper panel scale bar = 2 μM; lower panel scale bar = 0.5 μM. ^*^p < 0.05 vs MCAo, ^#^*p* < 0.05 vs tPA. T + S: (tPA + SSS)

To evaluate BBB permeability, we conducted Evans Blue (EB) leakage assay. As the data shown in Fig. 3A, B, the result revealed that delayed tPA administration exacerbated BBB leakage; As we expected, the combined administration of SSS and tPA remarkably reduced EB leakage in ipsilateral brain of AIS mice, with almost no Evans blue leakage. To evaluate the change of BBB phenotype, transmission electron microscopy (TEM) analysis was applied. Since BBB damage is strongly associated with brain hemorrhage, we assessed the extent of brain hemorrhage in each group of mice. By comparing the hemoglobin levels in brain homogenates of different groups, it showed a positive correlation between the extent of brain hemorrhage and the degree of BBB damage. We observed that tPA administration increased the extent of brain hemorrhage, whereas SSS, whether used alone or in combination with tPA, led to a nearly 75% reduction in the severity of brain hemorrhage (Fig. 3C). We conducted TEM to observe TJs in each group, as shown in Fig. 3D, the tight junctions in ipsilateral brain of mice in AIS group were disrupted, and the damage to tight junctions was even more severe in AIS mice received delayed tPA administration. In contrast, SSS supplement attenuated the deterioration. These results indicate that SSS treatment mitigates BBB permeability due to delayed tPA administration, preserving the integrity of BBB, contributing to the reduction of hemorrhagic transformation.

### SSS treatment ameliorated the disruption of tight junction proteins caused by delayed tPA administration

**Fig. 4:**
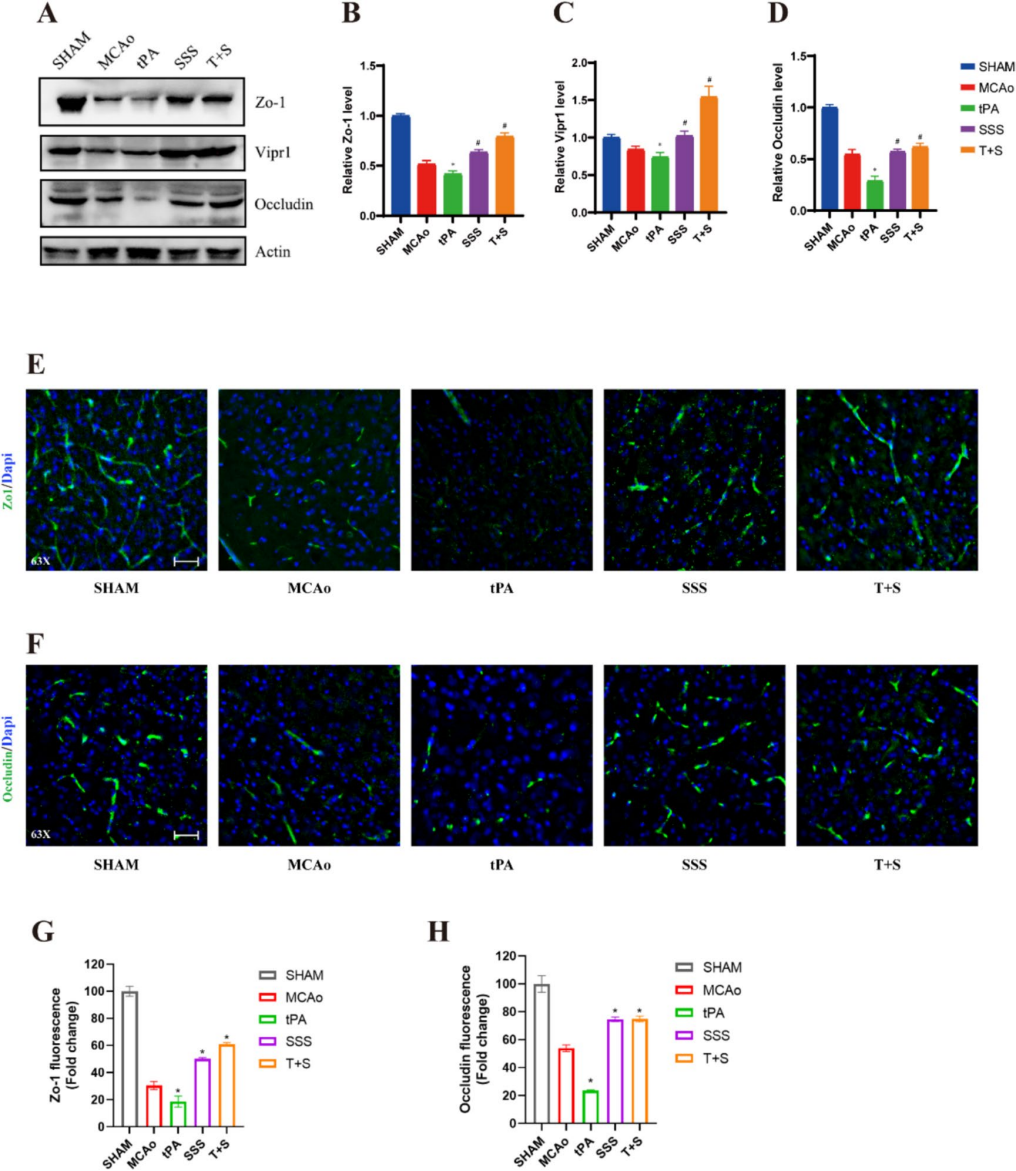
SSS treatment increased tight junction protein expression in AIS mice after delayed tPA administration. **A** Western blotting of Tight junction proteins ZO-1, VIPR1, Occludin. **B–D** Quantification analysis of expressions of ZO-1 (**B**), VIPR1 (**C**), Occludin (**D**). **E, F** Representative images of immunofluorescent staining of ZO-1 (**E**) and Occludin (**F**). **G–H** Quantification analysis of expressions of ZO-1 (**G**) and Occludin (**H**). scale bar = 25 µm. ^*^p < 0.05 vs MCAo, ^#^*p* < 0.05 vs tPA

By the detection of tight junction proteins expression, the results indicated the expressions of ZO-1 and Occludin in MCAo group were lower than those in the Sham group, and were worsened compared with those in tPA group by western blotting (Fig. 4A–D) and immunofluorescence staining (Fig. 4E–H). However, the combination treatment of SSS and tPA reversed the exacerbated trend due to the synergistic therapeutic effects.

### SSS treatment enhanced VIP and its receptor expressions after AIS

**Fig. 5:**
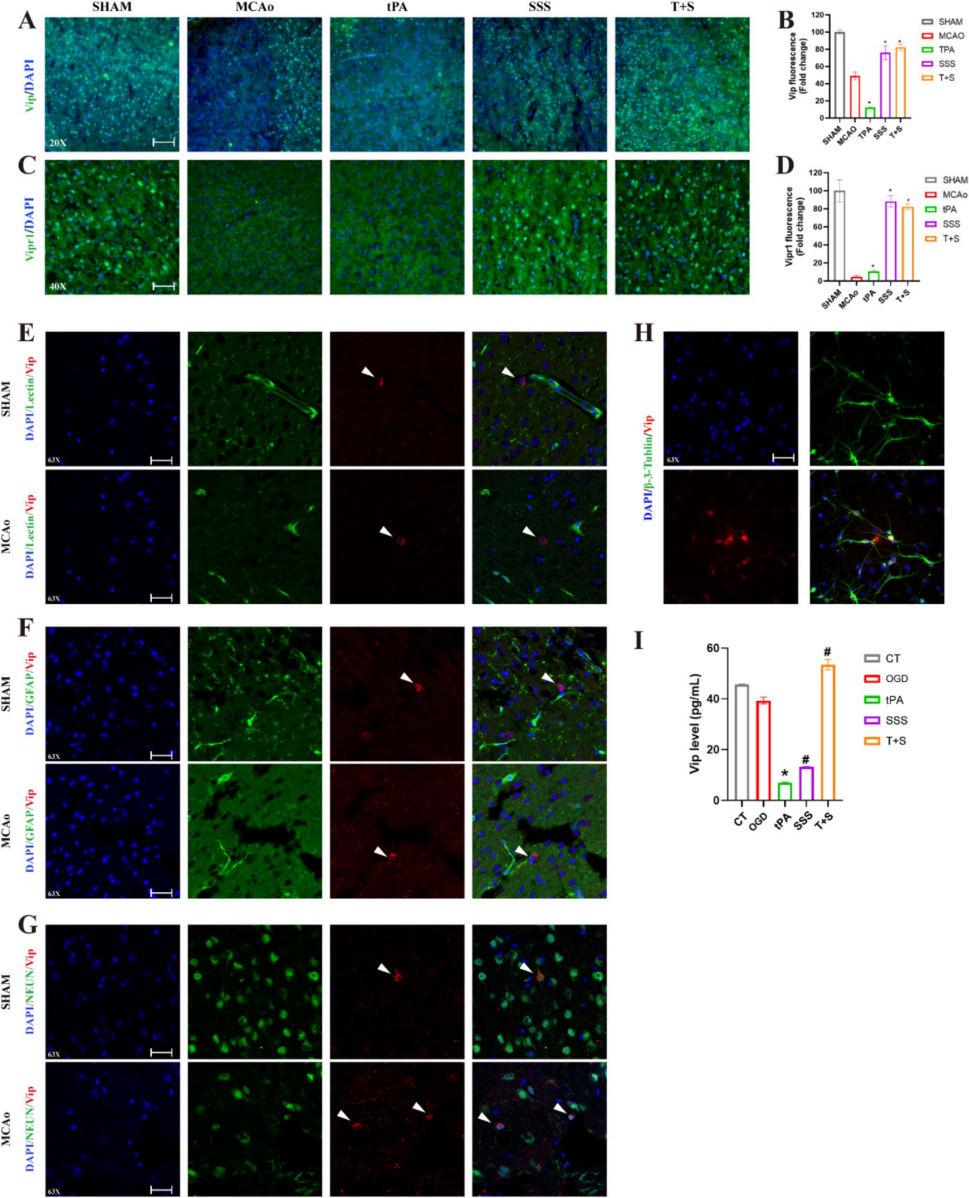
SSS treatment enhanced the expressions of VIP and VIPR1 after tPA administration. **A** Representative images of immunofluorescent staining of VIP. **B** Quantification analysis of expressions of VIP. **C** Representative images of immunofluorescent staining of VIPR1. **D** Quantification analysis of expressions of VIPR1. **E–G** Co-localization of VIP and Lectin (**E**), GFAP (**F**), NeuN (**G**).** H** Colocalization of VIP and primary neuron. **I** VIP quantified by Elisa assay. 20 × images scale bar = 100 µm; 40 × images scale bar = 50 µm; 63 × images scale bar = 25 µm. *p < 0.05 vs MCAo, ^#^*p* < 0.05 vs tPA

Based on our previous research, SSS maintained BBB integrity by enhancing VIP and its receptors in the brain post-stroke [25]. Therefore, we verified whether SSS treatment alleviated BBB damage resulted from delayed tPA administration by activating VIP/VIPRs pathway. The results suggested that VIP expression was decreased in MCAo group, and delayed tPA administration exacerbated this decline trend, while SSS treatment increased VIP expression, and the combined administrations with SSS and tPA further maintained the elevated trend (Fig. 5A, B). Simultaneously, VIPR1 expression had similar alteration (Fig. 5C, D).

In order to determine which kind of cells in brain exerted predominant role in VIP secretion, the immunofluorescence staining for VIP with the markers of endothelial cells, astrocytes, and neurons were conducted. As shown in Fig. 5E–G, it showed that the immunofluorescence of VIP with marker of neurons rather than the markers of endothelial cells and astrocytes was co-localized. To further confirm VIP secreted by neurons, we employed primary mouse neurons and detected VIP expression by immunofluorescence staining, and the results verified our hypothesis (Fig. 5H).

In vitro, tPA, SSS-containing serum and their combination were added into OGD insulted N2a cells, respectively. After 24 h, the cell supernatants were collected and ELISA assay was performed. It suggested that the combined administration of SSS and tPA significantly increased the secretion of VIP, although tPA administration inhibited VIP secretion (Fig. 5I). These findings demonstrated that VIP in the mouse brain was primarily expressed by neurons, and SSS treatment antagonized the deteriorated action resulted from tPA administration.

### SSS treatment protected the viability of OGD-insulted BMECs stimulated by tPA

**Fig. 6:**
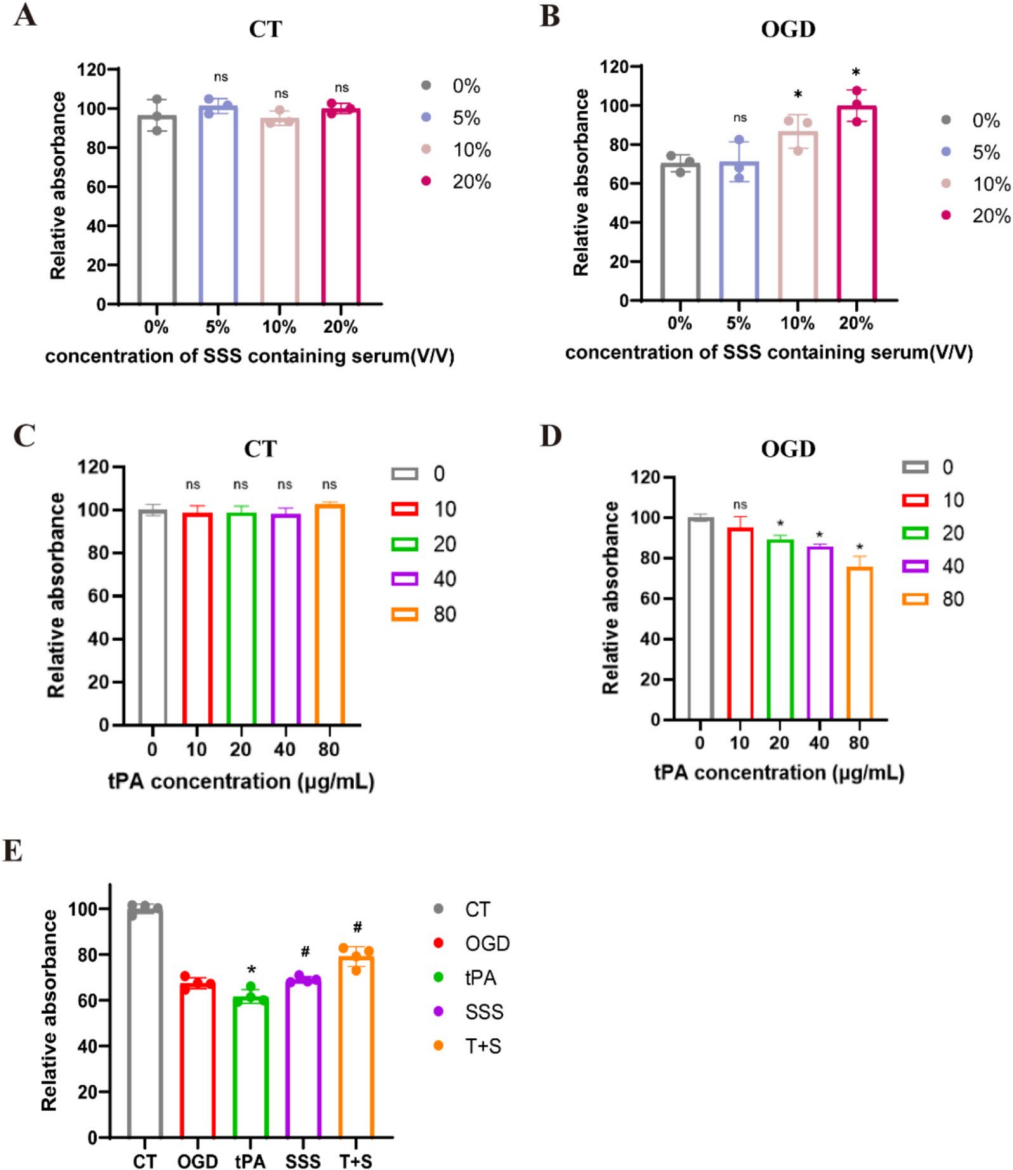
SSS containing serum improved OGD-insulted cerebral endothelial cells viability after tPA administration. **A** Toxicity evaluation of SSS containing serum on bEnd.3 cells under normal conditions by CCK8 assay. **B** The determination for optimal dose of SSS containing serum on OGD-insulted bEnd.3 cells. *p < 0.05 vs 0%. **C** Toxicity assessment of tPA on bEnd.3 cells under normal conditions. **D** The determination of toxic dose of tPA on OGD insulted bEnd.3 cells. *p < 0.05 vs 0 μg/mL. **E** The SSS containing serum protected the viability of OGD insulted bEnd.3 cells suffered from tPA. *p < 0.05 vs OGD; ^#^*p* < 0.05 vs tPA

In order to define the optimal dose of SSS-containing serum, we employed the concentrations of 5%, 10%, and 20% SSS-containing serum and there were no cytotoxic effects on the endothelial cell line bEnd.3. Subsequently, these three concentrations of SSS-containing serum were added to OGD-insulted bEnd.3 cells and it showed that the administrations of 10% and 20% SSS-containing serum exerted protective effects (Fig. 6A, B). Due to 10% SSS-containing serum already suggesting obviously protective efficacy, we selected this concentration for the next studies.

Next, to determine what dose of tPA caused BMECs injury, various doses of tPA were added into normal and OGD-insulted bEnd.3 cells, respectively. The results indicated that 80 μg/mL of tPA failed to affect bEnd.3 cells proliferation under normal conditions (Fig. 6C), while under OGD conditions, 20 μg/mL of tPA began to exhibit cytotoxic effects resulting in low viability of bEnd.3 cells (Fig. 6D). Moreover, the cytotoxicity of tPA under OGD conditions displayed a dose-dependent manner. Based on our results and report from another study [25], 20 μg/mL of tPA was regarded as the following administered dose for OGD insulted bEnd.3.

To investigate whether SSS treatment could protect OGD insulted bEnd.3 cells under tPA administration condition, cell viability was assessed using CCK-8. The results revealed that the administration of SSS-containing serum significantly enhanced the viability of OGD insulted bEnd.3 cells after tPA treatment (Fig. 6E).

### SSS treatment alleviated the worsening permeability of OGD-insulted BMECs incubated with tPA via VIP/VIPR1 pathway

**Fig. 7:**
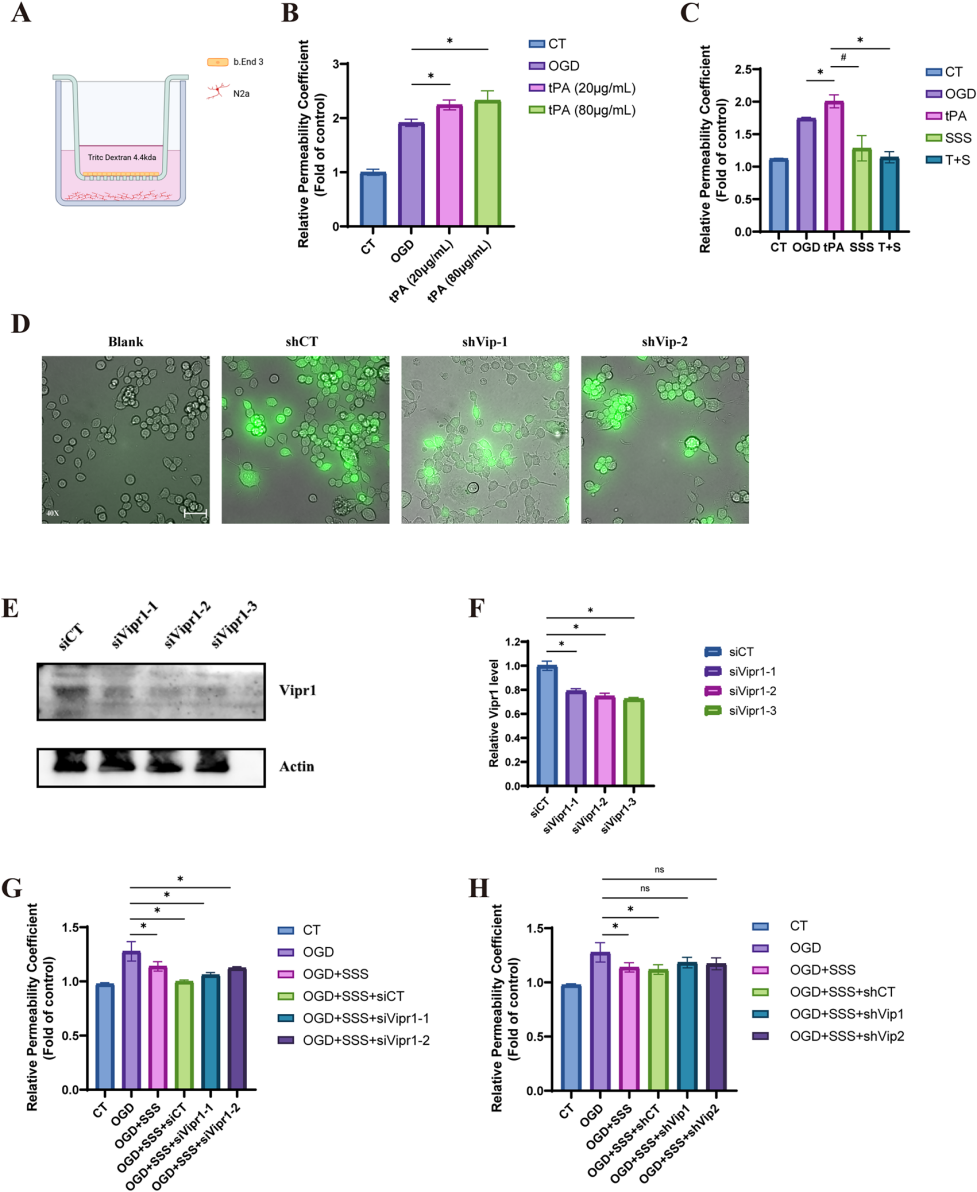
SSS treatment alleviated the worsening permeability of OGD insulted bEnd.3 cells barrier due to delayed tPA administration by VIP/VIPR1 pathway.** A** Schematic diagram of the co-culture of bEnd.3 cells and N2a. **B** tPA administration increased the permeability of OGD insulted bEnd.3 cells barrier. **C** SSS-containing serum treatment maintained the integrity of OGD insulted bEnd.3 cells barrier after tPA administration. **D** VIP knockdown in N2a cells verified by immunofluorescence. **E** VIPR1 knockdown in bEnd.3 cells verified by western blotting. **F** Quantification analysis of expressions of VIPR1 in bEnd.3 cells infected with VIPR1 siRNAs. **G** Knockdown of VIPR1 in bEnd.3 cells attenuated the protective effects of SSS-containing serum on OGD insulted bEnd.3 cells barrier injury resulted from tPA treatment. **H** Knockdown of VIP in N2a cells attenuated the protective effects of SSS-containing serum on OGD insulted bEnd.3 cells barrier injury resulted from tPA treatment. 40 × images scale bar = 50 µm *p < 0.05 vs MCAo

In order to discover the mechanism of SSS against the leakage of OGD-insulted bEnd.3 cells suffered from tPA, N2a and bEnd.3 cells were co-cultured in transwell (Fig. 7A). It suggested that tPA administration significantly increased the leakage of TRITC-dextran under OGD condition in a dose-dependent manner, displaying a deterioration of barrier integrity for bEnd.3 cells (Fig. 7B). SSS-containing serum administration reduced TRITC-dextran leakage, and also diminished the extent of barrier disruption when co-administered with tPA (Fig. 7C).

To verify if the therapeutic effects of SSS are related to VIP/VIPR1 pathway, we employed lentiviral shRNA to knock down VIP expression in N2a cells (Fig. 7D). Subsequently, we employed siRNA to knock down VIPR1 expression in bEnd.3 cells (Fig. 7E, F). It indicated that silencing VIPR1 in bEnd.3 cells compromised the efficacies of SSS on the permeability of BMECs (Fig. 7G). Similarly, silencing VIP in N2a cells diminished the protective effects of SSS on the integrity of OGD-insulted bEnd.3 cells suffered from tPA in vitro (Fig. 7H). These findings suggested that the mechanism of SSS on BMECs integrity was related to the activation of VIP/VIPR1 pathway.

### SSS contains multiple VIPR1 interactive compounds by examining the SSS-administered mice serum

**Fig. 8:**
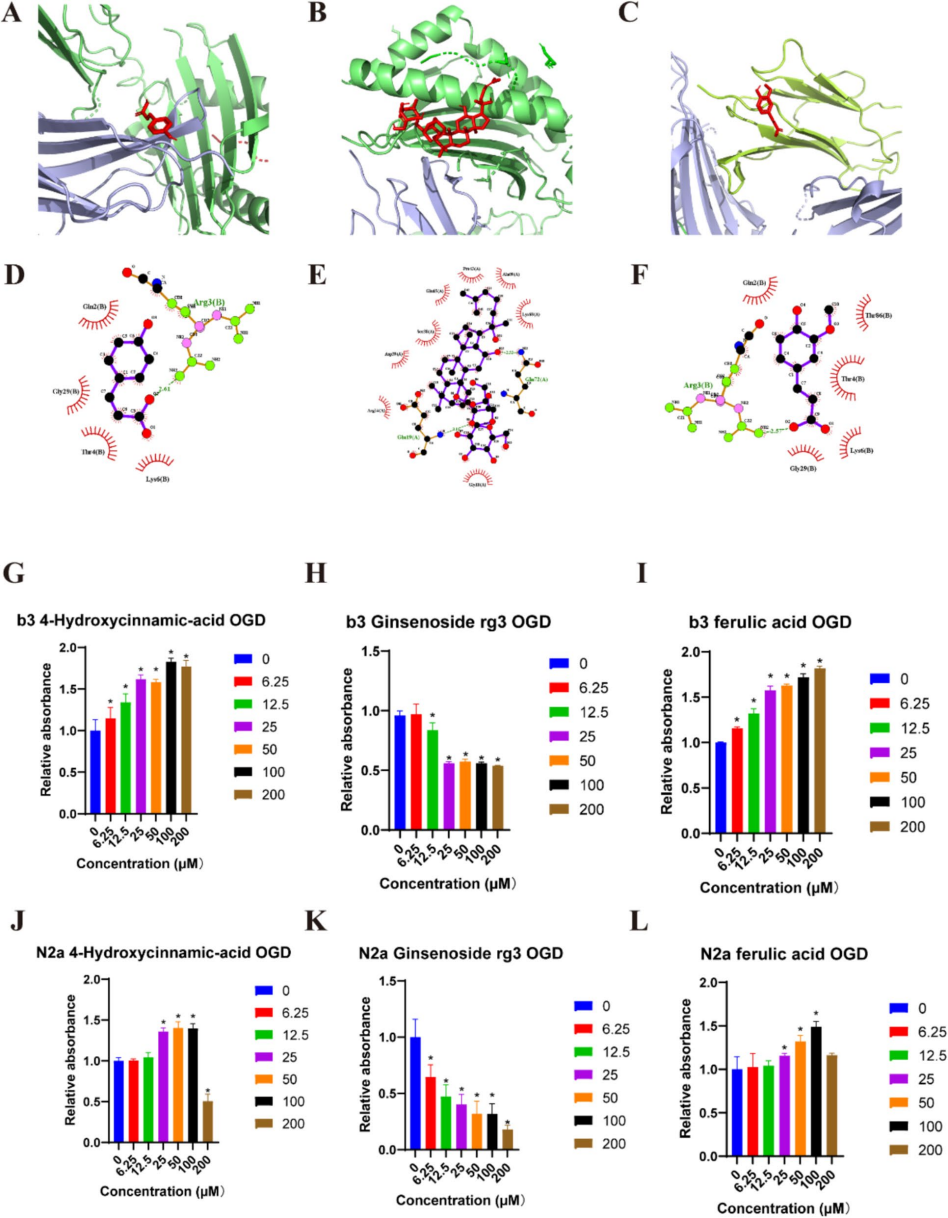
The cytotoxicity assessment of active components from SSS for normal and OGD insulted bEnd.3and N2a cells. **A–C** 3D-structural complex of VIPR1 bound with 4-Hydroxycinnamic acid (**A**), Ginsenoside rg3 (**B**), ferulic acid (**C**). **D–F** 2D-binding mode of VIPR1 bound with 4-Hydroxycinnamic acid (**D**), Ginsenoside rg3 (**E**), ferulic acid (**F**). **G–I** Protective effect of 4-Hydroxycinnamic acid (**G**), Ginsenoside rg3 (**H**), ferulic acid (**I**) on OGD insulted bEnd.3. **J–L** Protective effects of 4-Hydroxycinnamic acid (**J**), Ginsenoside rg3 (**K**), ferulic acid (**L**) on OGD insulted N2a. *p < 0.05 vs 0 μM

To explore the active compounds in SSS, we analyzed the bioavailable components which entered the serum. As summarized in Table S3, 4-Hydroxycinnamic acid, Ginsenoside Rg3, and Ferulic acid were identified as potential active compounds in SSS. Our results indicated that SSS protected the BBB permeability by stimulating VIP secretion. We hypothesized whether these compounds could not only stimulate VIP secretion in neuron cells but also directly bind to VIPR1 and activate the VIP/VIPR signaling pathway. The molecular docking analysis revealed binding energies of −5.11, −4.38, and −5.07 kcal/mol for 4-Hydroxycinnamic acid, Ginsenoside Rg3, and Ferulic acid, respectively, with VIPR1 (Fig. S2). These binding energies suggest that the ligands can form stable complexes with the receptor. The binding modes of these compounds with VIPR1 are shown in 3D (Fig. 8A–C) and 2D (Fig. 8D–F) representations. Based on these data, the active compounds in SSS may directly interact with VIPR1, leading us to investigate whether they could exert protective effects on cells subjected to OGD insult. Cell viability assays demonstrated that all three candidate compounds exhibited protective effects on bEnd.3 cells (Fig. 8G–I) and N2a cells (Fig. 8J–L).

## Discussion

Presently, despite extensive pharmacological studies on AIS, only tPA is approved by FDA for thrombolytic therapy. However, the employment of tPA beyond therapeutic time window leads to serious complications, particularly intracranial hemorrhage, which is especially concerned in AIS treatment [26]. Many traditional Chinese medicine formulas have protective effects on cerebral ischemic injury and are widely used in clinical in several Asian countries [27]. In our previous studies, we demonstrated that SSS, a classic Chinese medicine formula, had protective effects on BBB after cerebral ischemia [20]. Therefore, we hypothesize SSS treatment can extend therapeutic time window of tPA to exert synergistic efficacies and reduce the risk of hemorrhagic transformation resulted from BBB injury.

In the current study, when the thread was withdrawn in MCAo mice after 5 h and tPA was administered, it suggested that the delayed tPA treatment failed to exert therapeutic effects, instead of exacerbating neurological functional deficits. In contrast, the combination treatment of SSS and tPA ameliorated cerebral blood flow, infarction volume and neurological function, and reduced cerebral hemorrhage. Simultaneously, by Evans Blue staining, the results also demonstrated that delayed tPA administration worsened BBB leakage, and decreased BMECs' tight junction proteins expression, while the supplement of SSS reversed the trends. It suggested SSS treatment not only antagonized the adverse actions of delayed tPA administration, but also improved therapeutic effects on AIS treatment.

VIP is a peptide composed of 28 amino acids. It plays a crucial role in many physiological processes, including regulating blood flow and neural function [28, 29]. In previous studies, VIP, initially identified as a peptide predominantly funding in the small intestine, had been recognized for its significant role in maintaining intestinal barrier integrity. For instance, food intake rapidly activated neurons to produce VIP. Upon activation, these neurons selectively expressed the VIP receptor 2, which interacted with interleukin-22 (IL-22) in the intestine. This interaction leads to a reduction in antimicrobial peptide levels derived from epithelial cells, thereby preserving the dynamic balance between intestinal barrier integrity and nutrient absorption [18, 19]. In our previous studies, we found that SSS treatment exerted a protective effect on BBB through VIP [20]. Therefore, in this study, we verified whether SSS treatment activated the secretion of VIP in the brain after AIS, contributing to reducing BBB damage resulted from delayed administration of tPA. The immunofluorescence staining results indicated that the expression of VIP was highly co-localized with neurons rather than astrocytes or BMECs, which is consistent with its intrinsic nature as a neurotransmitter [30]. Thus, we speculated that SSS treatment maintained BBB integrity efficacy was related to the activation of VIP secretion from neurons.

By co-culture of N2a and bEnd.3 cells using a transwell system, our experimental results demonstrated that OGD-insulted bEnd.3 cells barrier permeability in tPA treatment group was inhibited by the administration of SSS-containing serum. However, when we knocked down VIP in N2a cells using lentivirus, the protective effects of SSS were compromised. Similarly, when we knocked down VIPR1 in bEnd.3 cells, the protective effects were also weakened. These results suggested that SSS treatment activated VIP in neuronal cells resulting in binding to VIPR1 on BMECs to maintain the integrity of BBB.

However, there are still limitations in this study. First, it remains unclear how SSS stimulates neuronal cells to secrete VIP and VIP/VIPR1 pathway regulated tight junction proteins, more detailed mechanism between neurons and BMECs should be figured out in future studies. Further research is required to define the downstream of VIPR1 for BBB protection after delayed tPA administration. By literature review, we found a possible pathway that may explain how VIP/VIPR1 pathway activation alleviates BBB breakdown. A study found that VIP treatment significantly increased Cyclic adenosine monophosphate (cAMP) contents in rat BMECs, leading to VEGF production, which could be blocked by a protein kinase A (PKA) inhibitor, suggesting that cAMP/PKA pathway could be activated by VIP/VIPR pathway [31]. Activation of PKA inhibited thrombin-induced phosphorylation of both myosin light chain (MLC) and the Myosin light chain phosphatase (MLCP) regulatory subunit myosin-targeting subunit 1 of myosin light chain phosphatase (MYPT1). Additionally, PKA activation also suppressed the RhoA/Rock pathway [32]. Another study revealed that activation of the ROCK/MLC signaling pathway led to early BBB disruption, primarily through sustained actin polymerization and the breakdown of junction proteins in microvascular ECs [33]. Together, the activation of the VIP/VIPR pathway activates the cAMP/PKA pathway, and subsequently inhibits the activation of the MLC/ROCK pathway, thereby protecting the integrity of the BBB. These reports may provide evidences of how VIPR1 contributes to BBB integrity.

Besides, a Chinese herbal medicine may contain hundreds or even thousands of components, not to mention SSS is a formula containing 3 herbal medicines. To better understand the mechanism of how SSS maintains BBB permeability, it is important to figure out the active compounds. Our molecular docking results show the candidate compounds can form a stable structure with VIPR1, which may stimulate the downstream VIP/VIPR1 pathways directly. The cell viability assay also supports our hypothesis, in which the active compounds treatment rescues the viability of OGD-insulted bEnd.3 cells and the protective effect is exhibited in a concentration-dependent manner. Nevertheless, next experiments should be conducted to investigate the individual contributions of these active compounds activating VIP/VIPR pathway and maintaining BBB integrity.

## Conclusion

Overall, in this study, SSS treatment either exerted antagonistic efficacies against the exacerbation of BBB permeability resulted from delayed administration of tPA, or combined with tPA to improve neurological function after AIS, and the mechanism of BBB protection was associated with the activation of VIP in neurons binding to VIPR1 on endothelial cells contributing to the maintenance of tight junction phenotype. Our study also suggests the potential advantages of SSS for the extension of tPA therapeutic time window in AIS treatment.

## Supplementary Information

Additional file 1.

## Data Availability

Data will be made available on reasonable request.

## Abbreviations

AIS: Acute ischemic stroke
MCAo: Middle Cerebral Artery Occlusion Model
BBB: Blood brain barrier
CBF: Cerebral blood flow
mNSS: Modified Neurological Severity Score
SSS: Shengui Sansheng San
tPA: Tissue plasminogen activator
VIP: Vasoactive intestinal peptide
VIPR1: Vasoactive intestinal peptide receptor 1
