# Supplementary material for: Shengui Sansheng San alleviates the worsening of blood–brain barrier integrity resulted from delayed tPA administration through VIP/VIPR1 pathway
Source: Chin Med. 2025 Mar 18;20:38. doi: 10.1186/s13020-025-01079-0 (PMC11916937; doi:10.1186/s13020-025-01079-0)
Supplement: Supplementary file 1 — Additional file 1. [file 13020_2025_1079_MOESM1_ESM.zip › New folder/New Microsoft Word Document.docx]

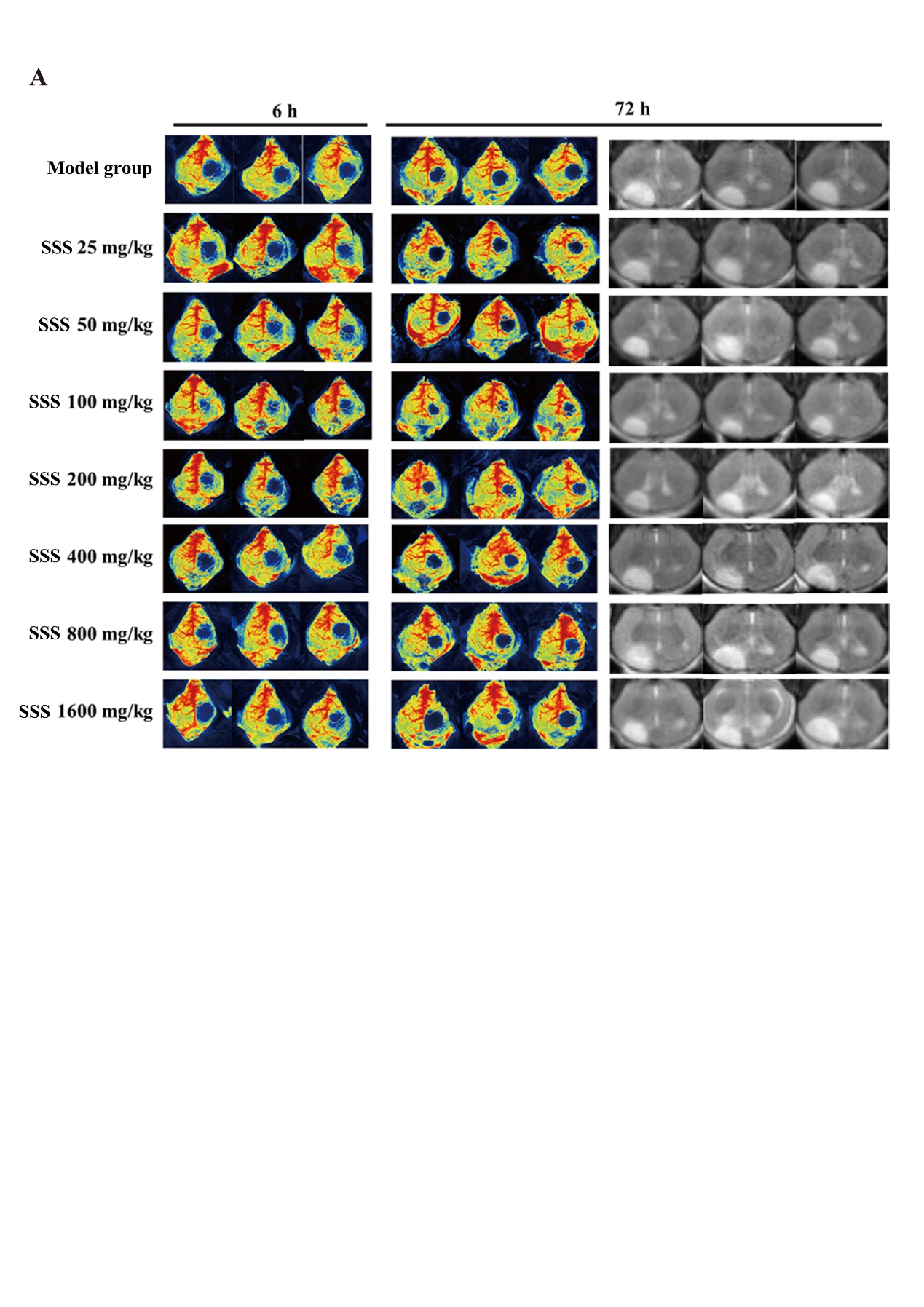


**Figure S1. The optimal dose determination of SSS.** LSCI and MRI images of different dosage of SSS administration in photochemical thrombosis induced mice after 6 and 72 hours.

**Photochemical-induced Stroke Model**

Male C57BL/6 mouse were subjected to focal cerebral ischemia through photothrombosis of cortical vessels. Cerebral infarction was induced by intravenous injection of rose bengal (20mg/kg) The mouse’s head was fixed in a stereotaxic apparatus, and the skull was exposed through a midline incision, cleared of the periosteum. Each hemisphere of the brain was irradiated with cold light at λ = 560 nm for 10 min.

**Assessment of infract volume by MRI imaging**

MRI imaging (1 T, T2-weighted, one-touch MRI) to assess infarct volume in stroke mouse was conducted 72 hours post-ischemic stroke onset under 2% isoflurane anesthesia by a small animal MRI system (NIUMAG Co. Ltd, China). Mouse were positioned on the MRI scanner at 37°C during the procedure. Prior to scanning, sagittal, coronal, and axial planes were confirmed. T2-weighted imaging was then performed with the following parameters: horizontal FOV 30, vertical FOV 30, horizontal resolution 0.25, vertical resolution 0.25, inter-slice gap 0.1, TR 3000 ms, TE 71.09 ms, ETL 12, and voxel size 0.25×0.25×2 mm. The entire process was completed within 7.5 minutes.


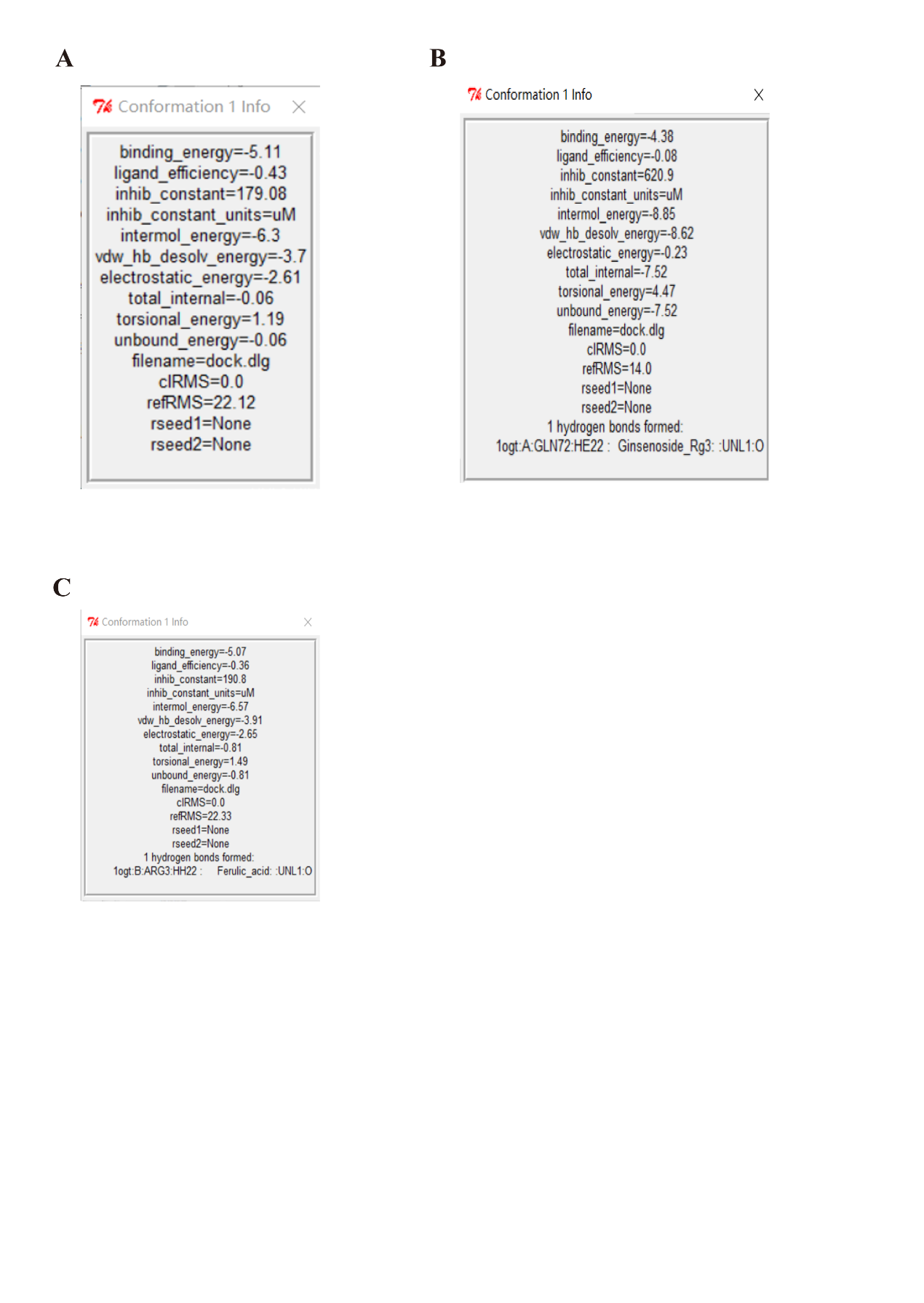


**Figure S2. Binding energy of SSS active compounds. (A)** Binding energy of 4-Hydroxycinnamic-acid and VIPR1 (-5.11 kcal/mol). **(B)** Binding energy of Ginsenoside-Rg3 and VIPR1 (-4.38 kcal/mol). **(C)** Binding energy of Ferulic-acid and VIPR1(-5.07 kcal/mol).

**Table S1. Modified neurological severity score (mNSS)**

| **Test** | Points |
| --- | --- |
| Motor tests |  |
| Raising the mouse by the tail | 3 |
| 1 Flexion of forelimb |  |
| 1 Flexion of hind limb |  |
| 1 Head moved more than 10 to the vertical axis within 30 s |  |
| Walking on the floor (normal = 0; maximum = 3) | 3 |
| O Normal walk |  |
| 1 Inability to walk straight |  |
| 2 Circling toward the paretic side |  |
| 3 Falling down to the paretic side |  |
| Sensory tests | 2 |
| 1 Placing test (visual and tactile test) |  |
| 2 Proprioceptive test (deep sensation, pushing the paw against the table edge to stimulate limb muscles) |  |
| Beam balance tests (normal = 0; maximum = 6) | 6 |
| 0 Balances with steady posture |  |
| 1 Grasps side of beam |  |
| 2 Hugs the beam and one limb falls down from the beam |  |
| 3 Hugs the beam and two limbs fall down from the beam, or spins on beam (>60 s) |  |
| 4 Attempts to balance on the beam but falls off (>40 s) |  |
| 5 Attempts to balance on the beam but falls off (>20 s) |  |
| 6 Falls off: no attempt to balance or hang on to the beam (>20 s) |  |
| Reflexes absent and abnormal movements | 4 |
| 1 Pinna reflex (a head shake when the auditory meatus is touched) |  |
| 1 Corneal reflex (an eye blink when the cornea is lightly touched with cotton) |  |
| 1 Startle reflex (a motor response to a brief noise from snapping a clipboard and paper) |  |
| 1 Seizures, mycolonus, myodystony |  |
| Maximum points | 18 |

**Table S2. SSS volatile oil extraction ratio**

| Order | SSS powder(g) | SSS volatile oil (g) | extraction ratio (%) |
| --- | --- | --- | --- |
| 1 | 233.51 | 0.89 | 0.38 |
| 2 | 280.87 | 1.57 | 0.56 |
| 3 | 287.05 | 2.09 | 0.73 |
| 4 | 336.23 | 2.55 | 0.76 |
| 5 | 323.43 | 2.55 | 0.66 |
| 6 | 314.68 | 2 | 0.64 |
| 7 | 336.95 | 2.1 | 0.62 |
| 8 | 332.16 | 2.67 | 0.80 |
| 9 | 339.81 | 2.3 | 0.68 |
| 10 | 384.82 | 2.63 | 0.68 |
| 11 | 396.19 | 3.06 | 0.77 |
| 12 | 403.2 | 3.12 | 0.77 |
| 13 | 413.85 | 3.3 | 0.80 |
| 14 | 404.98 | 3.12 | 0.77 |
| 15 | 390.81 | 3.04 | 0.78 |
| 16 | 382.41 | 3.2 | 0.84 |
| 17 | 365.75 | 2.85 | 0.78 |
| Total | 5926.7 | 42.64 | 0.72 |

**Table S3. Active compounds of SSS entering blood stream**

| NameEN | CAS | mzmed | rtmed | KBXQ (mean) | SSS_STW (mean) | SSS_SJ （mean） | SSS_STW vs SSS_SJ  blood_level |
| --- | --- | --- | --- | --- | --- | --- | --- |
| Ginsenoside Rg3 | 11019-45-7 | 783.49 | 465.10 | 0.00 | 7949293.14 | 94257.14 | I |
| trans-Ferulic acid | 537-98-4 | 193.05 | 294.50 | 2075809.71 | 679856180.03 | 8384194.58 | II |
| 4-Hydroxycinnamic acid | 7400-08-0 | 163.04 | 296.00 | 2690047.22 | 64086116.55 | 6878236.49 | II |

The candidate compounds were selected according to the difference between SSS_SJ vs KBXQ. KBXQ: blank serum; SSS_STW: mixture of SSS aqueous extract and volatile oil extract. SSS_SJ: SSS containing serum. The names, CAS registry number and mean peak score of each compound in indicated groups are listed.

**mzmed**: The median mass-to-charge ratio, representing the mass-to-charge ratio of this peak in all samples.

**rtmed:** The median retention time represents the retention time of this peak in all samples.

**I, II**: Blood entry condition classification levels: Level I represents: prototype components that enter the bloodstream; Level II represents: potential prototype components that may enter the bloodstream.
