# Supplementary figures and images for: Shengui Sansheng San alleviates the worsening of blood–brain barrier integrity resulted from delayed tPA administration through VIP/VIPR1 pathway
Source: Chin Med. 2025 Mar 18;20:38. doi: 10.1186/s13020-025-01079-0 (PMC11916937; doi:10.1186/s13020-025-01079-0)

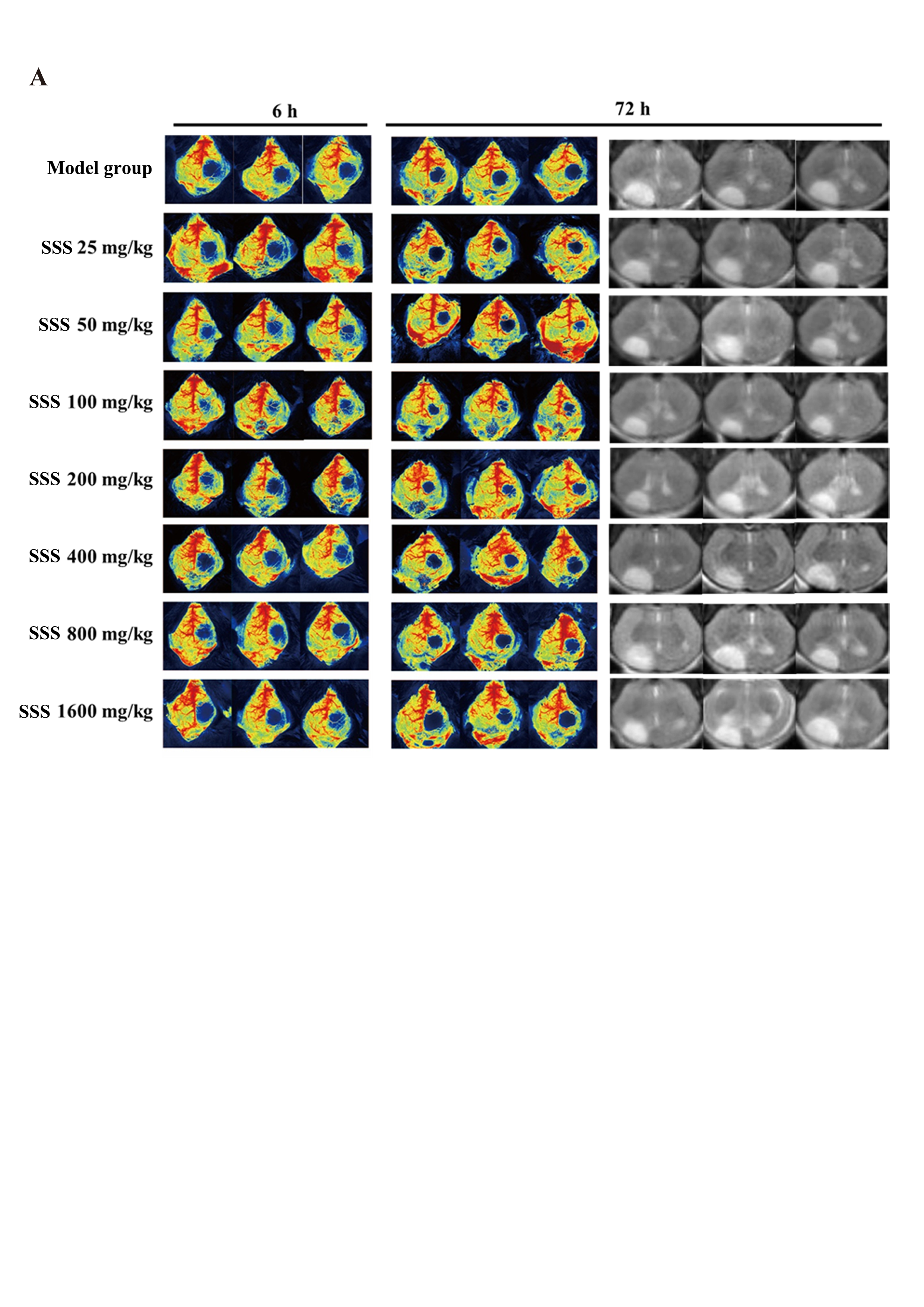

Supplement: Supplementary file 1 — Additional file 1. [file 13020_2025_1079_MOESM1_ESM.zip › New folder/Figure-S1.tif]

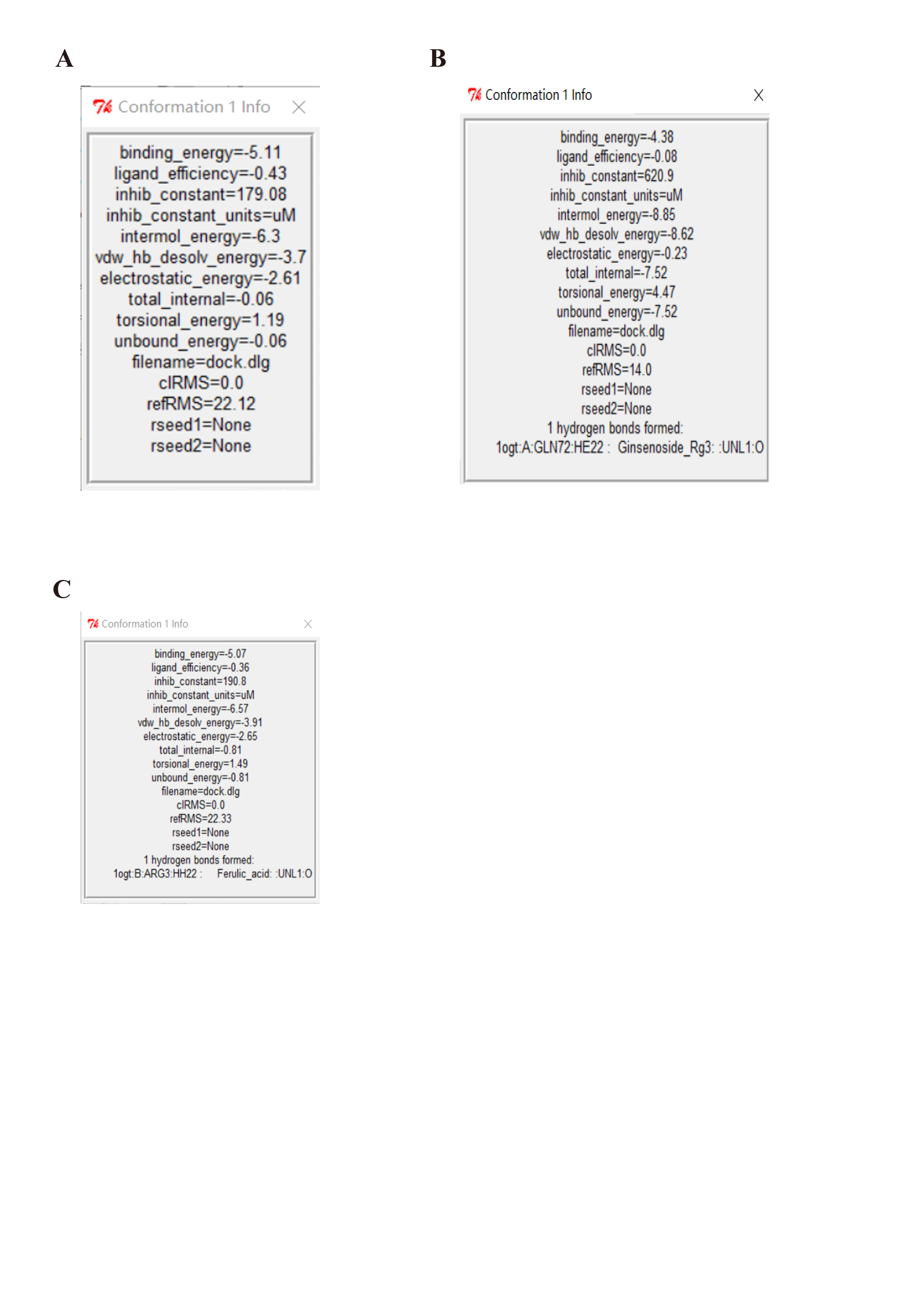

Supplement: Supplementary file 1 — Additional file 1. [file 13020_2025_1079_MOESM1_ESM.zip › New folder/Figure-S2.tif]
